# Low back pain, pelvic pain, and associated factors in type 1 diabetic pregnant women

**DOI:** 10.1016/j.clinsp.2024.100325

**Published:** 2024-02-07

**Authors:** Patricia Andrade Batista, Cláudia de Oliveira, Rafaela Alkmin da Costa, Rossana Pulcineli Vieira Francisco, Fabio Roberto Cabar

**Affiliations:** a Department of Obstetrics and Gynecology, Faculdade de Medicina, Universidade de São Paulo (USP), São Paulo, SP, Brazil; b Department of Physical Therapy, Universidade Santa Cecília (UNISANTA), Santos, SP, Brazil

**Keywords:** Diabetes mellitus type 1, Pregnant women, Low back pain, Pelvic pain

## Abstract

**Introduction:**

Pregnancy and diabetes mellitus promote several musculoskeletal changes predisposing this population to complaints of Lower Back (LB) and Pelvic Pain (PP).

**Objective:**

To assess the frequency of LB and PP and associated factors in type 1 Diabetic (DM1) pregnant women. Method: An observational analytical cross-sectional study. Thirty-six pregnant women with DM1 were evaluated through a postural assessment with a focus on pelvic positioning and what patients reported. The associated factors were assessed using the State-Trait Anxiety Inventory (STAI), the International Consultation on Incontinence Questionnaire Short Form (ICIQ-SF), and the Female Sexual Function Index (FSFI).

**Results:**

The frequency of LB and PP was 55.6 % and 30.6 %, respectively. The presence of anxiety was not associated with a higher prevalence of pain. The incidence of sexual dysfunctions was higher in the GD. DM1 duration had a mean of 14.9 years (± 8.2 SD) in the GD and 9.0 years (± 6.9 SD) in the GSD, which was statistically significant (*p* ≤ 0.050). In the multiple binary regression analysis for the occurrence of pain, the independent factor was DM1 duration ≥ 17 years (OR = 11.2; 95 % CI = 1.02‒124.75). The association between DM1 duration ≥ 17 years and being overweight showed a probability of 95 % for the studied population in the analysis of the probabilities of occurrence of the pain event.

**Conclusion:**

There was a high frequency of LB and PP related to pregnancy in DM1 pregnant women in the second trimester of pregnancy. The incidence of sexual dysfunction and DM1 duration ≥ 17 years increases the chance that DM1 pregnant women will experience pain. There was no association between anxiety. urinary incontinence and pain in DM1 pregnant women.

## Highlights

• DM1 is associated with changes in skeletal muscle functionality.
• During pregnancy, the incidence of low back pain and pelvic pain is moderate and the presence of DM1 may be associated with the early appearance of this symptom.
• The study demonstrated that pregnant women with DM1 complain of pain with a moderate incidence in the second trimester of pregnancy.
• Factors such as length of illness are related to the higher frequency of pain in pregnant women with DM1.

## Introduction

Type 1 Diabetes *Mellitus* (DM1) can manifest at any age, but it usually has its first manifestation and diagnosis before the age of thirty, being more frequent in school years and adolescence [1]. Available data indicate that 2 % to 5 % of all pregnant women are affected by DM and its maternal and fetal complications 2, 3, 4.

According to estimates, the prevalence of gestational low back and pelvic pain in pregnant women ranges from 30 % to 78 % 4, 5, 6, 7, 8, 9 and higher when there is a history of pelvic trauma, multiparity, chronic or pre-pregnancy low back pain, and when women are young [4,6,10, 11, 12].

The association between DM and neuromuscular repercussions has been widely studied, and the literature demonstrates that, due to the decrease of or damage to muscle mass, the loss of strength is one of the main functional changes observed [13,14]. During the gestational period, the skeletal muscle is the main target organ of glucose metabolism, and its contractile potential is directly altered by the action of progesterone [15].

Furthermore, women with DM1 have been shown to have higher serum levels of relaxin compared to non-diabetic women [16]. Therefore, a physiological association between DM and gestational low back and pelvic pain is expected [15].

The drug treatment of pain during pregnancy has restrictions on the use of analgesics [4,16]; the use of Non-Steroidal Anti-Inflammatory Drugs (NSAIDs), which during pregnancy is especially contraindicated in the third gestational trimester; and the use of opioids, which is not considered safe in this period [16]. Based on this, obstetric physiotherapy has been recommended as the first line of treatment for these painful manifestations during pregnancy [4,9].

The hypothesis of the present study is based on the possibility that DM1 causes functional, metabolic, and structural changes in the skeletal muscle system, reducing its functional capacity, which can be associated with the weakening and alteration of the muscular function of diabetic pregnant women. Therefore, these pregnant women could be more predisposed to developing low back pain and pelvic pain typical of the gestational period.

Thus, this study aimed to determine the frequency of low back and pelvic pain during pregnancy and to analyze their predictive factors in DM1 pregnant women in the second trimester.

## Methods

An observational and cross-sectional study was carried out with pregnant women who underwent prenatal follow-up in the endocrinopathies and pregnancy group of the endocrinopathy outpatient clinic of the Divisão de Clínica Obstétrica do Hospital das Clínicas da Universidade de São Paulo (HCFMUSP) from May 2016 to May 2019, in which 36 pregnant women with DM1 were assessed.

This study was approved by the Research Ethics Committee of the HCFMUSP under the number CAAE 1.552.712, approval date of 05/20/2016. The participants were informed about the study and signed an informed consent form according to Resolution 466/2012 of the National Health Council.

### Inclusion criteria

Pregnant women diagnosed with DM1; gestational age between 20 and 24 weeks, a period in which there is a physiological reduction of the pelvic floor muscles strength and a gradual stabilization of relaxin concentrations [17]; single fetus gestation; age between 18 and 37 years old at the time of admission to the study, which is considered the ideal age for the reproductive period [18] and all who had read, agreed, and signed the informed consent form.

### Exclusion criteria

Important orthopedic changes such as scoliosis or lower limb discrepancies and neurological antecedents cause a cognitive impairment or a motor deficit in the lower limbs.

The authors used convenience sampling to recruit participants who were patients in prenatal follow-up at the HCFMUSP. After they were included in this study, they were referred to an interview with the researching physiotherapist on the day of the routine consultation. Those who were not available to be interviewed on that day were scheduled for the day of the next prenatal consultation.

The pregnant women were assessed using an identification questionnaire, a postural assessment with a focus on pelvic positioning, a visual numerical pain scale, a graphic representation of the human body, and what patients reported. Associated factors were assessed using the State-Trait Anxiety Inventory (STAI), the International Consultation on Incontinence Questionnaire ‒ Short Form (ICIQ-SF), and the Female Sexual Function Index (FSFI).

The identification questionnaire gathered information including sociodemographic data such as age, gestational age, pre-gestational weight, current weight, height, education, marital status, profession, and health conditions including gestational history.

For the questions regarding gestational lower back and pelvic pain, the participants were asked about the presence of pain at some point during pregnancy and, if so, they pointed to the location of the pain on a representation map of the human body and classified the feeling of discomfort between 0 to 10 according to the numerical visual scale, with 0 being no pain and 10 being the strongest pain. After collecting sociodemographic and clinical data, the pregnant women's postures and pelvic positions were assessed to identify relevant orthopedic and neurological alterations that could predispose the subjects to pain.

The STAI was used to assess the level of anxiety. The instrument, validated in Brazil by Biaggio et al. [19] measures different concepts of anxiety: trait-anxiety (T-anxiety) and state-anxiety (S-anxiety) using two self-report scales. The T-anxiety scale is made up of 20 questions on how respondents feel, by ticking one of the alternatives: 1- Almost never; 2- Sometimes; 3- Often; 4- Almost always. On the A-state scale, also made up of 20 questions, they must indicate how they feel at the exact moment they are answering the questions, ticking one of the options: 1- Not at all; 2- Somewhat; 3- Moderately so; 4- Very much so. The scores of the two STAI scales range from 20 points to 80 points, being classified as “no or low anxiety” (20‒34 points), “moderate anxiety” (35‒49 points), “high anxiety” (50‒64 points), and “very high anxiety” (65‒80 points).

The International Consultation on Incontinence Questionnaire-Short Form (ICIQ-SF) was translated and validated into Portuguese by Tamanini et al. [20] and was used for Urinary Incontinence (UI) evaluation. In a simple, brief, and self-administered manner, the ICIQ-SF evaluates the frequency and amount of urine loss, the conditions of urine loss, the interference of this condition in activities of daily living, and the impact of UI on quality of life. The ICIQ-SF was chosen because of its suitability for Brazilian society. The questionnaire defines the results as follows: “no impact” (0), “light impact” (1‒3), “a moderate effect” (4‒6), “severe impact” (7‒9), and “very severe impact” (10 and above) [21].

The Female Sexual Function Index (FSFI) questionnaire was translated and validated for Brazilian Portuguese pregnant women by Leite et al. [22] was used. It can be self-administered by women who have had sexual activity in the previous four weeks. It consists of 19 questions that assess the female sexual response in six domains: sexual desire, sexual arousal, vaginal lubrication, orgasm, sexual satisfaction, and pain. The response options are scored between 0 and 5, increasingly, except for the questions about pain, in which the score is defined inversely. The total score is the sum of scores for each domain multiplied by the factor corresponding to each domain. The minimum score is 2 and the maximum score is 36 [17]. A total score less than or equal to 26 is considered a risk for sexual dysfunction.

As for data analysis, the SPSS Statistics software version 21.0 was used for the comparison of quantitative variables. The Student's *t*-distribution was used for parametric data and the Mann-Whitney *U* test for nonparametric data. The analysis between categorical independent variables associated with pain outcomes was performed using the chi-squared test or Fisher's exact test when the sample size of the analyzed variable was small. The binary logistic regression analysis was performed to identify the Odds Ratios (OR) and their respective 95 % Confidence Intervals (CI) between the independent variables and the pain outcome. The multiple linear regression analysis was used for variables with a *p*-value < 0.20 and those with clinical relevance in the univariate analysis. The method for including the variables in the multiple models was stepwise regression. The log-linear formula was used to calculate the probabilities. A descriptive level of 5 % (*p* ≤ 0.05) was assumed for statistical significance.

## Results

The sample analysis showed that the mean age of the 36 participants was 27.80 years (±5.59 SD). The average gestational age for the participants at study enrollment was 21.19 gestational weeks (±1.45 SD).

The clinical characteristics of the studied population show gestational history data in which 55.6 % (*n* = 20) of the participants were primiparous and 44.4 % (*n* = 16) were multiparous. Most pregnant women, 94.4 % (*n* = 34), had a stable partner. Regarding education levels, 47.2 % (*n* = 17) of the subjects had completed secondary school or had higher education. Regarding the BMI, the mean was 25.77 kg/m^2^ (± 3.58 SD), so most participants presented adequate weight. The mean disease duration was 13.03 years (± 8.17 SD).

Among the pregnant women studied, pre-pregnancy anxiety was present in only 18.2 % (*n* = 6) and 36.4 % (*n* = 12) had anxiety during the gestational period. Regarding the presence of urinary incontinence and sexual dysfunction, 44.1 % and 91.2 % of the pregnant women presented the complaints, respectively, during pregnancy.

In the group studied, 69.4 % (*n* = 25) of the participants reported low back or pelvic pain during pregnancy, constituting the pain group (GD). Six of these patients reported an association between low back and pelvic pain.

The participants who reported no pain formed the Group without Pain (GSD) (*n* = 11).

The frequency of gestational low back and pelvic pain was 55.6 % and 30.6 %, respectively, in DM1 pregnant women, who were in their second trimester. The mean low back pain score was 2.7 (± 2.7 SD), whereas the mean gestational pelvic pain score was 1.6 (± 2.6 SD). None of the patients scored 1 or 2 on the pain analogue scale.

When the correlation between sociodemographic and clinical variables of pregnant women in both groups was performed, only the duration of illness showed a statistically significant difference between them (*p* = 0.05), i.e., the longer the duration of illness, the more frequent the complaint of pain.

The analysis of the variables of interest between the GD and the GSD showed a general score related to sexual function of 21.6 (± 3.8 SD) for the GD and 18.9 (± 2.5 SD) for the GSD, with a statistically significant difference between the groups (*p* = 0.038) in such a way that pregnant women with complaints about pain had a greater change in sexual function. The variables anxiety and urinary incontinence did not show statistically significant differences between the groups (Table 1).

**Table 1:** Score of variables of interest in pregnant women in the groups with and without pain (*n* = 36) – HCFMUSP – May 2016 to May 2019.

**Variables**	**GD (*n* = 25)**			**GSD (*n* = 11)**			**p**
	**n**	**Average (±SD)**	**Median**	**n**	**Average (±SD)**	**Median**	
			**(p25‒p75)**			**(p25‒p75)**	
**STAI T-anxiety score**	23	46.3 (±5.8)	46.0 (42‒49)	10	42.6 (±6.2)	42.0 (39‒44)	0.103
**STAI S-anxiety score**	23	47.5 (±47.0)	47.0 (42‒54)	10	47.1 (±7.0)	47.5 (40‒50)	0.861
**ICIQ-SF score**	23	4.2 (±5.1)	3.0 (0‒5)	11	2.5 (±4.7)	0.0 (0‒6)	0.363
**FSFI score**	23	21.6 (±3.8)	21.0 (19‒24)	11	18.9 (±2.5)	18.0 (17‒21)	0.038^a^

a Student's *t*-distribution.

The disease duration variable showed a statistically significant association in the univariate binary logistic regression analysis for the pain outcome. DM1 pregnant women with ≥ 17 years of disease had an 11-fold chance of having pain when compared to those with less than 17 years of disease (OR = 11.0; 95 % CI = 1.01‒120.43).

During the multiple binary regression analysis for the pain outcome, the independent factor for the outcome, corrected for BMI, was the disease duration ≥ 17 years (OR = 11.2; 95 % CI = 1.02‒124.75) (Table 2).

**Table 2:** Multiple linear regression analysis of pain in type 1 diabetic pregnant women – HCFMUSP – May 2016 to May 2019.

**Variables**			**95 % CI**		**p**
	**β (EP)**	**OR_adjusted_**	**Minimum**	**Maximum**	
Intercept	0.108 (0.68)				
Duration of DM1^a^ (≥17 years / < 17 years)	2.423 (1.23)	11.27	1.02	124.75	0.048^b^

Model adjusted by BMI values.

a Variable stratified in tertiles.

b p < 0.05 – statistically significant.

When analyzing the probabilities of the pain event occurring, it is noteworthy that DM1 pregnant women with a disease duration equal to or over 17 years and being overweight have a probability of 0.95 or 95 % of having pain (Table 3).

**Table 3:** Analysis of the probability of the pain event occurring in type 1 diabetic pregnant women – HCFMUSP – May 2016 to May 2019.

**Situation**	**Duration of the disease**	**BMI**	**The probability of the event occuring**
1	< 7 years	Normal range	0.53
2	< 7 years	Overweight	0.61
3	7 to 16 years	Normal weight	0.61
4	7 to 16 years	Overweight	0.69
5	≥17 years	Normal weight	0.93
6	≥17 years	Overweight	0.95

## Discussion

The present study was carried out to analyze the frequency of low back and pelvic pain in DM1 pregnant women in the second trimester of pregnancy and its association with their level of anxiety, presence of sexual dysfunction, and urinary incontinence. Possible predictors of pain in the studied population were also verified.

In a literature review, DM1 and the gestational period were associated with a higher frequency of musculoskeletal changes, which may be related to greater pain complaints [10]. Initially, this study assessed the frequency of low back and pelvic pain during pregnancy in 36 diabetic pregnant women in the second trimester.

A study with 404 pregnant women, by Manyozo et al. [4], showed that 173 participants were in their second trimester, and 60 % of them reported gestational low back and pelvic pain. In the present study, 69.4 % of pregnant women with DM1 reported low back and pelvic pain during pregnancy in the second trimester, demonstrating that, in the population of this study, the frequency of the complaint was higher than in pregnant women with usual risk, i.e., low risk. This finding can be elucidated with the study by Steinetz et al. [14], which demonstrated that women with DM1 had higher serum levels of relaxin when compared to non-diabetic women. The literature has already described the association of high levels of relaxin with the development of pain in the pelvic girdle during pregnancy 23, 24, 25, 26.

Fruscalzo et al. [5] demonstrated in their study that the presence of low back pain increases significantly in frequency and intensity throughout pregnancy and was associated with more cesarean section during labor. In the present study, the incidence of low back pain was high, specifically in Brazil, this is relevant data for reducing cesarean sections.

The 2013 Cochrane systematic review showed moderate scientific evidence that acupuncture or exercises adapted to the stage of pregnancy are effective in the prevention and treatment of low back and pelvic pain during pregnancy [27]. Van Benten et al. [28] also demonstrated that the association of exercises with patient education has a positive effect on pain management.

Therefore, if pregnant women with DM1 were referred for physiotherapeutic follow-up in the first trimester of pregnancy or when they presented the first symptoms of discomfort, physiotherapists would be able to, through individualized exercises, provide better pain management and quality of life, and can reduce the chance of cesarean section because pain.

In the study by Egan et al. [29], the level of anxiety in 32 pregnant women with DM1 was moderate to severe in 37.5 % of the volunteers. This information corroborates the present study, which demonstrated a moderate anxiety rate in 36.4 % of its patients.

Considering the population of this study, the moderate level of anxiety could be justified by the fact that 20 of the 36 pregnant women were nulliparous; thus, the physical and emotional changes typical of pregnancy could have contributed to the verified anxiety index. Additionally, socioeconomic status and weekly prenatal consultations have a financial impact, which could also corroborate the level of anxiety. Moreover, for DM1 pregnant women, difficulties with glycemic control during pregnancy can be considered a variable that interferes with the anxiety level of this population.

The quality of sexual life of pregnant women with gestational low back and pelvic pain was studied by Mogren [30]. The survey showed that 30.7 % of pregnant women with mild to moderate pain reported less sexual satisfaction during pregnancy and among pregnant women with moderate to severe pain, the prevalence of dissatisfaction with their quality of sexual life was 38 %. The present study showed that the presence of sexual dysfunction was reported by 87 % of pregnant women who had gestational low back pain, pelvic pain, or a combination of both.

This study revealed that the disease duration of 17 years or more and being overweight were predictive factors for the presence of low back and/or pelvic pain during pregnancy.

However, no studies were found in the literature that related the presence of pain to the duration of the disease in pregnant women with pre-existing diabetes. Studies by Marini et al. [31] and Piculo et al. [32] demonstrated that DM interferes with the functionality of the skeletal striated muscles. Furthermore, they are the main target organ for glucose metabolism during pregnancy [13]. Thus, pregnant women in this study who had had the disease for 17 years or more could have had greater interference from the DM etiology in the functionality of their skeletal striated muscles, justifying the higher frequency of pain in this population.

The literature demonstrates that there is no consensus regarding the association between pain and weight gain during pregnancy. Nacir et al. [33], Mohseni-Bandpei et al. [34], and Sencan et al. [35], in their studies, found no significant difference between the BMI and the presence of pain in pregnant women. Beber and Satilmis [36], in their study with 400 pregnant women, when comparing women with and without low back pain, showed that the presence of pain was more prevalent in women who had gained more weight during pregnancy.

The presence of pain in pregnant women with DM was already described in the literature, as in the study by Eberhard-Gran and Eskild [12], which found a higher frequency of pain in obese diabetic pregnant women.

However, this study was the first to evaluate the frequency of pain in Brazilian pregnant women with DM1. Most studies in pregnant women with diabetes refer to gestational diabetes.

The available scientific studies show that pregnant women with gestational diabetes have a higher frequency of musculoskeletal disorders due to changes in the structure of muscle fibers caused by the pathophysiology of the disease.

The authors might think that pregnant women with DM1, by the time they become pregnant, will have been exposed to these changes for years and can present a higher frequency and even the onset of complaints earlier than that described in pregnant women at usual risk or with gestational diabetes.

This study was the first to evaluate the frequency of pain and UI in Brazilian pregnant women with type 1 diabetes. Most studies on pregnant women with diabetes refer to gestational diabetes. The aim of this study is to fill the gap in scientific knowledge regarding this important topic, as there are no studies that address the population of Brazilian pregnant women with pre-gestational diabetes.

As in other studies, the present research had limitations such as sample size as DM1 is a low prevalence condition, around 1 % to 2 %, during pregnancy; lack of monitoring of these patients, which, if monitored, would make it possible to verify the impact of these changes at more advanced gestational ages; frequency of these symptoms in the pre-gestational period was not evaluated, which could explain the increased frequency of complaints; and the fact that the authors did not find studies with a population similar to this research, which made comparisons of the findings impossible.

Obstetric physiotherapy plays a fundamental role in the prevention and treatment of musculoskeletal disorders and can be a great ally in improving the quality of life of DM1 pregnant women.

Based on the results of this study, physiotherapy could be recommended for pregnant women with DM1 in the first trimester to prevent these dysfunctions, which already have an increased frequency in this population starting in the second trimester, not only improving the quality of life for this population but also possibly contributing with better perinatal outcomes.

Therefore, this research suggests that new studies be proposed to verify whether the evolution of pregnancy implies a higher frequency of the assessed complaints; to investigate whether physiotherapy, through kinesiotherapy, could help prevent and minimize reported complaints; and to analyze whether physical therapy monitoring during pregnancy could contribute to better perinatal and postpartum outcomes.

## Conclusion

The frequency of low back and pelvic pain during pregnancy was high in the studied population, which was DM1 women in the second trimester of pregnancy. The incidence of sexual dysfunction in DM1 pregnant women and the disease duration equal to or greater than 17 years was associated with the presence of pain. There was no association between anxiety urinary incontinence and pain in DM1 pregnant women.

This observational study follows the STROBE Statement. Clinical Trial registration number is not applicable because this is an observational study.

## CRediT authorship contribution statement

**Patricia Andrade Batista:** Conceptualization, Methodology, Funding acquisition, Validation, Formal analysis, Data curation, Writing – review & editing, Writing – original draft. **Cláudia de Oliveira:** Conceptualization, Methodology, Validation, Formal analysis, Data curation, Writing – review & editing. **Rafaela Alkmin da Costa:** Writing – review & editing. **Rossana Pulcineli Vieira Francisco:** Conceptualization, Methodology, Validation, Formal analysis. **Fabio Roberto Cabar:** Conceptualization, Methodology, Validation, Formal analysis.

## Conflicts of interest

The authors declare no conflicts of interest.
